# The Association between Preoperative Vitamin D Levels and Postoperative Complications in Patients Undergoing Colorectal Liver Metastasis Surgery

**DOI:** 10.3390/jcm13010115

**Published:** 2023-12-25

**Authors:** Ahmad Mahamid, Esther Kazlow, Ariel Matan David, Omar Abu-Zaydeh, Aasem Abu Shtaya, Dvir Froylich, Wissam Khoury, Eran Sadot, Riad Haddad

**Affiliations:** 1 Department of Surgery, Carmel Medical Center, Haifa 3436212, Israel; mahamidam@yahoo.com (A.M.); ekazlow@gmail.com (E.K.); m.david@campus.technion.ac.il (A.M.D.); oabuzaydeh@gmail.com (O.A.-Z.); dvirfr7@gmail.com (D.F.); wekhoury@gmail.com (W.K.); 2 The Ruth and Bruce Rappaport Faculty of Medicine, Technion, Israel Institute of Technology, Haifa 3200003, Israel; asemab@clalit.org.il; 3 Department of Gastroenterology, Carmel Medical Center, Haifa 3436212, Israel; 4 Department of Surgery, Rabin Medical Center, Petch Tikvah 4941492, Israel; eransadot@gmail.com; 5 Sackler School of Medicine, Tel Aviv University, Tel Aviv 6997801, Israel

**Keywords:** colorectal liver metastasis, liver resection, vitamin D, complications

## Abstract

(1) Background: Over the past several years, there has been a renewed interest with regard to the effect of pre-operative vitamin D levels on post-surgical outcomes. Pre-operative vitamin D deficiency has been associated with many negative post-operative outcomes. However, the role of vitamin D in postoperative outcomes in colorectal liver metastasis (CRLM) resection is relatively uninvestigated. Our study investigated the correlation between preoperative vitamin D levels and postoperative complications in patients undergoing resection for CRLM. (2) Methods: We retrospectively examined the post-operative course of 109 patients, who were evaluated based upon preoperative vitamin D levels: the first group had vitamin D levels less than 25 nmol/L (VIT D < 25 nmol/L) (*n* = 12) vs. the second group who had vitamin D levels equal to or greater than 25 nmol/L (VIT D ≥ 25 nmol/L) (*n* = 97). (3) Results: Patients with lower pre-operative vitamin D levels (VIT D < 25 nmol/L) had significantly higher rates of blood transfusions (33.3% vs. 10.3%, *p* = 0.01), post-operative surgical complications (50% vs. 17.5%, *p* = 0.009), and infectious complications (25% vs. 7.2%, *p* = 0.04). However, there was no difference in overall survival seen between the two groups. (4) Conclusions: The results of our study indicate that patients with preoperative vitamin D deficiency (defined as preoperative vitamin D levels less than 25 nmol/L) may have an increased risk of postoperative complications in patients undergoing liver surgery for metastatic colorectal cancer.

## 1. Introduction

According to the World Health Organization (WHO), colorectal cancer is the third most prevalent cancer worldwide, and the second most common cause of cancer-related deaths. Almost half of all patients with colorectal cancer develop metastatic disease over the course of their illness, with the majority of metastases localized to the liver. For patients with colorectal liver metastases, surgical resection remains the most successful treatment, offering the best chance for survival [1].

The fortification of milk with vitamin D in the early 1930s was effective in substantially decreasing the prevalence of rickets and osteomalacia worldwide [2]. However, at the turn of the 21st century, the transition to a mainly indoor lifestyle, as well as the popularization of diets with highly processed foods, as well as an increase in sun avoidance, led to a reoccurrence of subclinical vitamin D deficiency in the modern world [3].

Vitamin D is a prohormone that must be metabolized into biologically active metabolites that can subsequently initiate a cascade of diverse physiologic and metabolic processes [4]. There are two main ways that vitamin D enters the body; through direct consumption in the diet or via synthesis in the skin from 7-dihydrocholesterol following UVB exposure. After entering the body, vitamin D is metabolized by vitamin D-25-hydroxylase (CYP2R1 & CYP27A1) in the liver, which hydroxylates vitamin D into the major circulating form, 25-hydroxyvitamin D (25(OH)D), also known as calcidiol. Because 25(OH)D is the major circulating form of vitamin D in the serum, it is typically used to determine a patient’s vitamin D status [5]. Then, 25(OH)D is converted into the fully active metabolite 1,25(OH)_2_D, also known as calcitriol, in the kidney via the enzyme 1-α-hydroxylase (CYP27B1) [6].

Initially, CYP27B1 was believed to be found solely in the kidney. However, in recent years, research has shown widespread presence of both vitamin D receptor (VDR) and CYP27B1 in tissues throughout the body including the immune system. In addition to the well-known effects of vitamin D on musculoskeletal health, there is growing evidence that vitamin D plays a critical role in helping the host repel both bacterial and viral infections. The mechanism of vitamin D’s activity includes production of cytokines, antimicrobial proteins, and pattern recognition receptors [6].

The protective role of vitamin D in preventing cancer was evaluated in ecological and observational studies which revealed reduced levels in cancer mortality in areas with higher sun exposure compared to areas with low sun exposure [7,8]. Recent studies have shown vitamin D to play a central role in many anti-inflammatory and immunomodulatory effects in the body. There is increasing evidence that vitamin D metabolites have the potential to promote cell differentiation and apoptosis and inhibit cancer cell proliferation and angiogenesis [9,10]. Deleterious effects of vitamin D deficiency have been reported in surgical critical care outcomes; these include excess mortality, longer length of hospital stay, higher incidence of sepsis, and longer periods of mechanical ventilation [11]. Additionally, there is growing evidence that vitamin D replacement therapy can improve post-surgical outcomes [12]. Several studies have identified pre-operative vitamin D status as the most relevant predictor of long-term post-surgical outcomes. A number of studies have shown that that peri-operative or post-operative vitamin D supplementation shows few benefits with regard to ultimate surgical outcomes, although this remains somewhat controversial [13,14]. Serum 25(OH)D is the preferred marker for assessing vitamin D status [15,16]. A level of <25 or <30 nmol/L (or 10/12 ng/mL) is consistent with severe vitamin D deficiency, and dramatically increases the risk of excess mortality, infections, and many other diseases [17,18]. Maintaining vitamin D levels above 30 nmol/L (or 12 ng/mL) is advised for public health purposes [19]. Our study, for the first time, examines the benefits of having sufficient vitamin D levels as compared to vitamin D deficiency with regard to post-surgical outcomes for patients undergoing liver surgery for CLRM.

## 2. Materials and Methods

Patients who underwent liver resection for CRLM between December 2004 and January 2019 were identified from the surgical databases at Carmel Medical Center (Haifa, Israel) and Rabin Medical Center (Petach Tikvah, Israel). Our review included examination of patient demographics, detailed surgical history, pathology results, and follow-up from the medical/oncology teams. The study was conducted in accordance with the Declaration of Helsinki and Good Clinical Practice Guidelines, and was approved by the institutional review board (IRB) of Carmel and Rabin Medical Centers. The pre-operative workup of all patients included standard blood tests, tumor markers, imaging modalities (computed tomography [CT], positron emission CT [PET-CT] and magnetic resonance imaging [MRI]), characterization of the specific tumor (number, location, size, and relation to intrahepatic vascular or biliary structures), and calculation of the future liver remanent volume. All patients were evaluated for surgery by an attending anesthesiologist at the respective institutions and approved for surgery by a multidisciplinary team. All patients were informed about the procedure, including risks and benefits. Written consent was obtained from all patients before undergoing surgery.

In our study we defined vitamin D deficiency as having serum 25(OH)D levels of 25 nmol/L or less. The vitamin D levels were retrospectively collected from the patients’ medical files. Only patients who did not receive perioperative vitamin D supplementations were included. Metastasis was defined as the presence of a liver tumor at the time of diagnosis or at follow-up in patients with colorectal cancer. Blood loss was defined as the volume of blood suctioned from the abdominal cavity during the surgery. Operative time was defined as the time elapsed from the initial incision until closure. Postoperative hospital stay was defined as the number of hospitalized days from the day of operation until the day of discharge, inclusive. We used the Clavien–Dindo grading system to characterize post-operative complications occurring within 30 days of surgery. Tumor size and resection margins were determined based upon pathology reports taken from the permanent sections of tissue samples. R0 was defined as an absence of cancer cells seen microscopically at the resection margin. After discharge, the patients were followed by our multidisciplinary team during the first month post surgery, every 4 months for the first 2 years, and subsequently twice a year. Follow-ups included clinical examinations, blood work-up including carcinoembryonic antigen (CEA), and spiral CT of the chest–abdomen or PET-CT as indicated.

### Statistical Analysis

All statistical analyses were performed using IBM statistics (SPSS 24, IBM, Armonk, NY, USA). Continuous variables were summarized with mean ± SD or median and IQR, as appropriate. Categorical variables were presented as numbers and proportions. Disease-free survival (DFS) and overall survival (OS) were estimated using Kaplan–Meier curves and compared between groups by log-rank test. *p* < 0.05 was considered statistically significant.

## 3. Results

**Figure 1:**
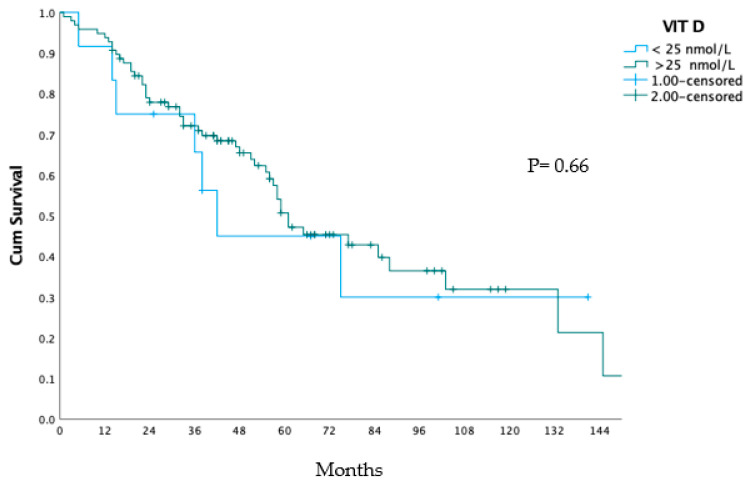
Overall survival after liver surgery.

Data from Table 1 and Table 2, and Figure 1 provide a detailed analysis of the differences and similarities between patients who underwent liver surgery for CRLM, as categorized by their preoperative vitamin D levels. The study included 109 patients who underwent liver surgery for metastatic colorectal cancer. Of these, 12 patients had preoperative vitamin D levels less than 25 nmol/L (VIT D < 25 nmol/L), and 97 patients had vitamin D levels equal to or greater than 25 nmol/L (VIT D ≥ 25 nmol/L).

Table 1 shows the baseline demographic, liver, and colorectal characteristics, as well as the perioperative and histological outcomes of the two groups. There was no significant difference between the groups regarding age (*p* = 0.09), gender (*p* = 0.15), BMI (*p* = 0.54), T-stage (*p* = 0.13), N-stage (*p* = 0.82), DFS (*p* = 0.82), clinical risk factor (Fong) (*p* = 0.59), size of largest liver tumor (*p* = 0.37), number of liver metastases (*p* = 0.75), perioperative chemotherapy (*p* = 0.92), or type of liver resection (*p* = 0.17), or sequence of resection (*p* = 0.97).

However, patients with a vitamin D level ≤ 25 nmol/L had a significantly higher rate of blood transfusion (33.3% vs. 10.3%, *p* = 0.011) and total complications (50% vs. 17.5%, *p* = 0.009) compared to the patients with a vitamin D level ≥ 25 nmol/L. Patients with a vitamin D level of ≤25 nmol/L had a higher rate of infectious complications compared to those patients with a vitamin D level ≤ 25 nmol/L group (25% vs. 7.2%, *p* = 0.04).

Table 2 shows the survival rates of the two groups at different points in time after liver surgery. The VIT D < 25 nmol/L group had lower survival rates compared to the VIT D ≥ 25 nmol/L group at all time points, and lower median overall survival (42 vs. 61 months, respectively). However, this difference was not statistically significant (*p* = 0.66). Figure 1 shows the overall survival curves of the two groups, which also did not show a significant difference (*p* = 0.66).

## 4. Discussion

The results of our study indicate that preoperative vitamin D deficiency may be associated with an increased risk of postoperative complications in patients undergoing liver surgery for CRLM. We found that patients with preoperative vitamin D levels less than 25 nmol/L had a higher rate of total complications, including the need for more blood transfusions, as well as increased incidence of infections compared to those with vitamin D levels ≥ 25 nmol/L.

Our findings align with the existing literature concerning the role of preoperative vitamin D deficiency and postoperative complications. A 2021 study conducted at the University of Health Sciences, Turkey, involved 104 patients undergoing colorectal cancer surgery. The patients were stratified based on preoperative vitamin D levels, using a threshold of 20 nmol/L. Researchers in this study observed a higher risk of infection in patients with lower vitamin D levels, an outcome that appeared independent of other variables [20]. Despite utilizing a slightly lower vitamin D deficiency threshold, this study parallels our observations and extends the relevance of vitamin D levels across various surgical contexts.

In addition to colorectal surgeries, the significance of vitamin D levels on post-operative outcomes has been highlighted in other surgical procedures. Data from the Humana Database between 2007 and 2016 revealed that vitamin D deficiency in patients undergoing total knee arthroplasty was associated with higher incidences of surgical site infection requiring irrigation and debridement, removal of the prosthetic joint, and other postoperative complications, such as deep venous thrombosis and myocardial infarction [21]. Furthermore, a research study focusing on cardiac surgery reported an inverse relationship between preoperative vitamin D levels and surgical site infections, further emphasizing the broader implications of vitamin D status in various surgical contexts [22].

The etiology of the observed association between preoperative vitamin D levels and increased risk of postoperative infections, as found in our study and other related research, could possibly be explained by examining vitamin D’s role in modulating the innate immune system.

Vitamin D enhances the production of antimicrobial peptides such as defensin β2 and cathelicidin, in addition to strengthening physical barriers such as epithelial cells. Additionally, vitamin D’s influence on increasing phagocytosis and chemotaxis aids in the rapid response to infection [23]. These immunomodulatory functions may explain the correlation found between vitamin D deficiency and increased risks in various surgical contexts, including colorectal, and, in our study, liver resection for metastatic colorectal cancer. Further research may provide deeper insights into these mechanisms and help assess the potential benefits of vitamin D supplementation in preoperative care for patients undergoing these surgeries.

Additionally, vitamin D’s role in cardiovascular health may have significant implications in the preoperative context, particularly for surgeries like liver resection for metastatic colorectal cancer. Studies have identified a connection between vitamin D deficiency and heart-related conditions such as heart failure (HF), arterial hypertension, and atherosclerosis [24]. These links are thought to be mediated through vitamin D’s influence on the renin–angiotensin system, endothelial function, inflammation, and calcium metabolism [25]. However, the relationship between vitamin D and cardiovascular health is complex, with research yielding both supportive and conflicting results. This complexity underscores the importance of careful consideration of vitamin D’s status in preoperative care, as it may impact patient outcomes.

Interestingly, we did not observe a significant difference in survival rate between the two groups in our study. This finding is somewhat surprising, as vitamin D has been shown to have anticancer properties [4], and has been associated with improved overall survival in patients with colorectal cancer. In a large-scale prospective analysis based on the UK Biobank, involving over 500,000 individuals aged 37–73 years, serum 25(OH)D concentrations were found to be inversely associated with CRC incidence. Additionally, increased pre-diagnostic concentrations of serum 25(OH)D were linked to improved CRC survival, especially in proximal colon cancers [26]. These findings highlight the complex nature of vitamin D’s role in cancer survival and incidence.

The lack of significant difference in survival rates between the two groups could possibly be explained by the diminishing influence of Vitamin D in metastatic disease. In the early stages of cancer, vitamin D’s anticancer properties could play a pronounced role. However, as the disease advances to a metastatic state, the influence of vitamin D might become overshadowed by a myriad of other genetic, environmental, and therapeutic factors. This complex interaction with other factors may create a dilutional effect, rendering the impact of vitamin D less significant in later stages of the disease, thus complicating its role in cancer survival and incidence.

While our study provides important insights into the relationship between vitamin D and surgical outcomes in patients with metastatic colorectal cancer, there are several limitations that should be noted. First, our sample size was relatively small, which may have limited our ability to detect significant differences in survival rates between the two groups. It also restricted our ability to carry out a subcategorical analysis for other perioperative variables. Second, we did not measure vitamin D levels postoperatively, so it is unclear how deficiency may have affected long-term outcomes. Finally, our study was retrospective in nature, which means that it is subject to the inherent biases and limitations of this study design. We believe that a multi-center prospective randomized control trial or using the propensity score matching method in a larger cohort will be the ideal study design for further analyzing the association between preoperative vitamin D levels and postoperative complications in patients undergoing colorectal liver metastasis surgery, and validating our results while including other confounders. Despite the limitations of our study, it may induce other researchers to further investigate and shed light on this interesting area and eventually become one of the cornerstones of changing clinical practice; this change will be induced by screening patients for vitamin D deficiency and providing vitamin D supplementation in the preoperative setting in order to reduce the morbidity rates after liver resection of CRLM.

## 5. Conclusions

Our study suggests that preoperative vitamin D deficiency may be associated with an increased risk of postoperative complications, particularly blood transfusion and infectious complications in patients undergoing colorectal liver metastasis resection. There was no significant difference in survival rates between the two groups. Further studies with larger sample sizes and prospective designs are needed to confirm these findings, and to investigate the potential post-operational benefits of vitamin D supplementation in this patient population.

## Figures and Tables

**Table 1:** Baseline demographic, liver, and colorectal characteristics, perioperative and histological outcomes.

	VIT D < 25 nmol/L (Total =12) N (%)	VIT D ≥ 25 nmol/L (Total = 97) N (%)	*p* Value
Age (liver surgery)	60 ± 11	66 ± 12	0.09
Gender			0.15
Male	3 (25%)	44 (45.5%)	
Female	9 (75%)	53 (54.5%)	
BMI	27.5 ± 3.6	26.4 ± 4.8	0.54
T			0.13
1	1 (8.3%)	2 (2%)	
2	3 (25%)	6 (6.2%)	
3	6 (50%)	60 (61.8%)	
4	1(8.3%)	9 (9.3%)	
unknown	1 (8.3%)	20 (20.5%)	
N			0.82
N0	5 (41.7%)	32 (33%)	
N1	5 (41.7)	29 (29.9%)	
N2	1 (8.3%)	16 (16.5%)	
unknown	1 (8.3%)	20 (20.6%)	
DFS (liver mets)			0.82
<12 months	6 (50%)	51 (52.6%)	
≥12 months	5 (41.7%)	37 (38.1%)	
unknown	1 (8.3%)	9 (9.3%)	
Clinical risk factor (Fong)			0.59
0–2	8 (66.7%)	67 (69.1%)	
3–5	3 (25%)	17 (17.5%)	
Unknown	1 (8.3%)	13 (13.4%)	
Size of largest liver tumor			0.37
<5 cm	10 (83.3%)	85 (87.6%)	
≥5 cm	2 (16.7%)	8 (8.2%)	
Unknown		4 (4.2%)	
Number of liver metastases			0.75
1	7 (58.3%)	57 (58.8%)	
>1	5 (41.7%)	40 (41.2%)	
Peri-operative chemotherapy			0.92
Yes	9 (75%)	71 (73.2%)	
No	3 (25%)	26 (26.8%)	
Blood transfusion			0.011
Yes	4 (33.3%)	10 (10.3%)	
No	5 (41.7%)	72 (74.2%)	
Unknown	3 (25%)	15 (15.5%)	
Liver resection			0.17
Anatomic	3 (25%)	12 (12.4%)	
Non-anatomic	9 (75%)	85 (87.6%)	
Sequence of resection			0.97
Liver first	1 (8.3%)	9 (9.3%)	
Colon first	9 (75%)	72 (72.2%)	
Combined	2 (16.7%)	18 (18.6%)	
Complication: total			0.009
Yes	6 (50%)	17 (17.5%)	
No	6 (50%)	19 (82.5%)	
Complication: infection			0.04
Yes	3 (25%)	7 (7.2%)	
No	9 (75%)	90 (92.8%)	

**Table 2:** Survival after liver surgery.

	VIT D < 25 nmol/L (Total = 12)	VIT D ≥ 25 nmol/L (Total = 97)	*p*
12 Months	83.3%	93.8%	
24 Months	75%	79%	
36 Months	65.6%	72.1%	
60 Months	45%	50.7%	
120 Months	30%	31.9%	
Median (95%CI) months	42 (31:53)	61 (45:77)	0.66

## Data Availability

Data are contained within the article.
